# Toxicology of Airborne Inorganic Arsenic: Oxidative Stress, Molecular Mechanisms, and Organ-Specific Pathologies

**DOI:** 10.3390/toxics13090753

**Published:** 2025-09-04

**Authors:** Qingyang Liu

**Affiliations:** College of Ecology and Environment, Nanjing Forestry University, Nanjing 210037, China; qyliu@njfu.edu.cn

**Keywords:** airborne inorganic arsenic, toxicological mechanisms, risk assessment, carcinogen

## Abstract

Arsenic, a naturally occurring metalloid, poses a significant global public health threat due to widespread environmental contamination. Despite its well-documented carcinogenicity, critical gaps remain in understanding the health impacts of chronic low-level airborne exposure and the multi-modal mechanisms driving inorganic arsenic toxicity. This narrative review synthesizes recent molecular research and population health data to explain how airborne inorganic arsenic causes harm through multiple biological pathways. Key novel insights include (1) a comprehensive analysis of inorganic arsenic-induced oxidative stress and epigenetic dysregulation, revealing transgenerational effects via germline epigenetic markers; (2) a critical evaluation of the linear no-threshold (LNT) model, demonstrating its overestimation of low-dose risks by 2–3× compared to threshold-based evidence; and (3) descriptions of mechanistic links between inorganic arsenic speciation, organ-specific pathologies (e.g., neurodevelopmental impairments, cardiovascular diseases), and pollution mitigation strategies. This study connects molecular mechanisms with public health strategies to improve arsenic risk assessment. It focuses on how inorganic arsenic alters gene regulation (epigenetics) and combines exposure from multiple sources, while also clarifying uncertainties about low-dose effects and refining safety standards.

## 1. Introduction

Arsenic (As), a naturally occurring metalloid with an average crustal abundance of 1.5–2 mg/kg, exhibits complex environmental behavior due to its diverse chemical speciation [1,2]. Inorganic arsenic (As^III^ and As^V^), organic compounds (e.g., methylarsonic acid), and volatile species like arsine (AsH_3_) participate in intricate biogeochemical cycles, influencing arsenic’s distribution and mobility [3,4,5]. While inorganic species are notoriously toxic, organic forms such as arsenobetaine in seafood are rapidly excreted and considered benign. Hence, subsequent references to ‘arsenic’ specifically denote its toxic inorganic forms unless stated otherwise [3]. Airborne arsenic emissions originate from both natural sources (e.g., volcanic eruptions, releasing ~7900 tons annually) and dominant anthropogenic activities, including metal smelting, coal combustion, and informal e-waste recycling, which collectively contribute to an estimated 24,000 tons of arsenic per year [6,7,8]. Notably, unregulated e-waste dismantling in Southeast Asia and South Asia has become a major pollution hotspot, releasing highly bioavailable arsenic nanoparticles [9].

Although arsenic exposure via drinking water has been extensively characterized, the risks of airborne inhalation remain underappreciated [10]. Inhalation accounts for less than 1% of total ingested arsenic mass intake, with higher pulmonary bioavailability (30% vs. 60% gastrointestinal absorption) in most populations. Occupational exposure (e.g., smelter workers) can elevate total arsenic levels in the lungs by 10–100-fold compared to the general public [11]. Critically, arsenic persists in lung tissue with a biological half-life of up to six years, facilitating chronic accumulation and a decades-long latency period for malignancies [12]. The International Agency for Research on Cancer (IARC) classifies inorganic arsenic as a Group 1 human carcinogen, with robust evidence linking it to lung, bladder, and skin cancers [13]. However, fundamental questions remain regarding low-dose, long-term exposure effects and nonlinear dose–response relationships [14,15]. This review addresses these gaps by synthesizing environmental monitoring data, molecular toxicology, and epidemiological studies from 2000 to 2025. Unlike previous reviews, which have focused on aqueous arsenic or individual toxicity pathways [2,11], this work provides a recent synthesis of airborne arsenic’s unique exposure dynamics, molecular mechanisms, and regulatory implications. Our objectives aim to (1) characterize global concentration distributions and transformation rules of atmospheric arsenic, (2) deconstruct multimolecular toxicity mechanisms, emphasizing metabolic activation, oxidative stress, and epigenetic regulation, (3) assess limitations of prevailing health risk assessment models, and (4) discuss mechanism-based prevention and control strategies. By tracking environmental pathways and deciphering cellular toxicity cascades, I aim to bridge the disconnect between risk models and real-world health effects, particularly at low doses, and propose precision public health interventions.

## 2. Methods

### 2.1. Search Strategy

A systematic literature search was conducted using the PubMed and Web of Science databases for publications from 1 January 2000 to 1 June 2025. Search terms included ‘airborne arsenic,’ ‘toxicity mechanisms,’ and ‘risk assessment,’ restricted to English-language studies. Inclusion criteria encompassed sample size (>20), experimental controls, and statistical rigor.

### 2.2. Quality Assessment

Eligible studies were required to report direct measurements of ambient total arsenic concentration, molecular toxicology assays (e.g., in human cell lines or animal models), or epidemiological analyses of ambient arsenic exposure. Only peer-reviewed journal articles and reports were considered. Exclusions involved studies with (1) fewer than 10 participants; (2) a lack of original data or reliance on secondary analyses; or (3) simulated exposures in molecular toxicology or epidemiological contexts.

## 3. Levels of Airborne Arsenic

### 3.1. Global Distribution

**Figure 1:**
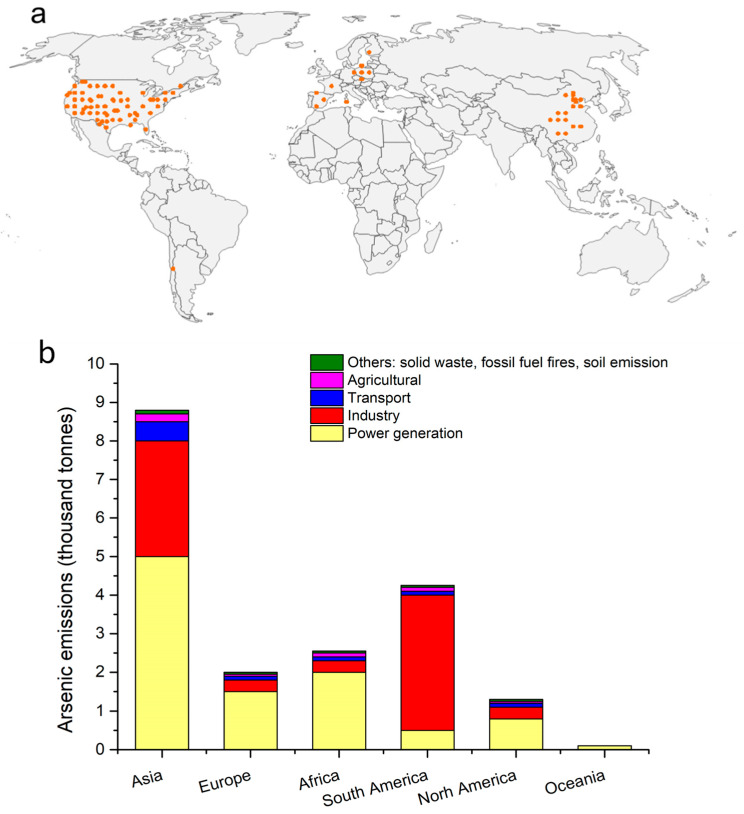
Global distribution and emission sources of airborne arsenic. (**a**) The observed sites of atmospheric arsenic from multiple sources, including the European Monitoring and Evaluation Programme (EMEP; http://ebas.nilu.no, accessed on 1 January 2025) for Europe, the Interagency Monitoring of Protected Visual Environments (IMPROVE; https://vista.cira.colostate.edu/Improve/, accessed on 1 January 2025) for the U.S., and supplementary regional studies [17]. The orange dots denote the sampling sites observed in the study (**b**) Emissions of atmospheric arsenic across different areas worldwide, as interpreted in Zhang et al., (2020) [17]. The spatial distribution of atmospheric arsenic concentrations was simulated using the GEOS-Chem model (v11-02), driven by Modern-Era Retrospective analysis for Research and Applications version 2 (MERRA-2) meteorological data at 104 4° × 5° resolution with 72 vertical layers using observational data at multiple sites. Model results and observational details are further documented in Zhang et al., (2020) [17]. Copyright (2020) National Academy of Sciences.

The global distribution of airborne arsenic is characterized by spatial heterogeneity, primarily dictated by the intensity of pollution sources, meteorological conditions, and geographical features [16]. Background concentrations in pristine remote regions, such as Antarctica, can be as low as 0.02 ng/m^3^, reflecting natural baselines unaffected by anthropogenic activities [17]. In contrast, rural areas influenced by agricultural practices (e.g., historical pesticide use) and residential coal combustion exhibit concentrations ranging from 3 to 200 ng/m^3^ [17]. Figure 1a lists the observed sites of atmospheric arsenic from multiple sources [17]. Airborne arsenic concentrations fluctuate seasonally. In the Northern Hemisphere, winter concentrations can be 2–3 times higher than summer levels due to increased coal use for heating, potentially elevating cumulative exposure risks [17]. This distribution is complicated by seasonal variation, particularly in the Northern Hemisphere. Winter concentrations frequently exceed summer levels by a factor of 2–3, a phenomenon largely attributable to increased coal combustion for heating purposes, which consequently elevates cumulative exposure risks [17]. In addition, toxic effects of short-term peak exposure (e.g., smelting plant accidents) may differ fundamentally from long-term low-concentration exposure, necessitating the development of dynamic risk assessment models [18,19,20].

Arsenic concentrations in industrial hotspots have increased by several orders of magnitude (Figure 1b) [21,22]. As the world’s largest copper producer, Chile had an average atmospheric arsenic concentration of 8 ng/m^3^ in 2015, with levels exceeding 100 ng/m^3^ in the northern copper mining belt [17,23]. In China’s eastern industrial zones (e.g., the Yangtze River Delta and Pearl River Delta), the annual average concentration of arsenic in PM_2.5_ is 4 ng/m^3^, but in specific industrial areas like the Shanxi coal and power base, peak concentrations can reach approximately 20 ng/m^3^, exhibiting values thousands of times higher than those found at an Antarctic research station [23,24,25]. Mining operations, even for non-arsenic-containing minerals, contribute substantially to human arsenic exposure, especially through inhalation. Studies near Arizona mining and smelting sites [26] found arsenic in aerosols in two different particle sizes: fine particles (~0.3 μm) from smelting emissions and coarse particles (~7.0 μm) from windblown tailings. Arsenic levels surged when winds carried smelter pollutants, increasing inhalation risks. Near the Iron King Mine Superfund site [27], airborne arsenic contaminated soils and crops, compounding exposure risks. In the Navajo Nation, abandoned uranium mines raised arsenic levels in water sources (e.g., 10–50 µg/L) [28], and the Gold King Mine spill [29] exposed communities to arsenic in river sediments and dust. India faces significant arsenic pollution risks, particularly from coal combustion and industrial activities [18]. Studies indicate that atmospheric arsenic levels near coal-fired power plants in regions like West Bengal can reach 50–120 ng/m^3^, far exceeding the concentrations found by the Antarctic research station [23,30]. Unregulated coal use during 2005–2015 contributed to marked increases in airborne arsenic, though precise national-scale quantification remains limited [18]. In certain regions of Chile, atmospheric arsenic levels have reached alarming concentrations of up to 30 ng/m^3^, posing serious threats to both air quality and public health due to extensive mining operations [17]. Airborne arsenic pollution in Europe and North American shows highly localized patterns [18]. Monitoring data from 2017 to 2018 indicated that only 1% of monitoring stations (7 out of 645) in 27 EU countries exceeded the annual average target of 6 ng/m^3^ [18,31]. Even with advanced flash smelting technology, arsenic concentrations in this area continue to exceed regulatory standards, highlighting limitations of existing pollution control technologies [32].

### 3.2. Chemical Forms

The environmental behavior and biological toxicity of airborne arsenic are intrinsically linked to its chemical speciation [10]. Inorganic arsenic (As^III^ and As^V^) exhibits biological toxicity, while organic species like arsenobetaine in seafood are non-toxic due to rapid excretion in urine [3,10]. Atmospheric arsenic primarily exists in inorganic forms, including arsenite (AsO_3_^3−^) and arsenate (AsO_4_^3−^) adsorbed onto fine particulate matter (PM_2.5_) [31]. Under oxidative conditions, As^III^ can be gradually oxidized to As^V^ by atmospheric oxidants like ozone and hydroxyl radicals over a period of hours or days [32]. However, the rate of this transformation on particle surfaces is influenced by factors such as pH and metal ion catalysis, often resulting in incomplete conversion [17].

Organic arsenic compounds, including methylarsonic acid (MMA) and dimethylarsinic acid (DMA), have been detected in atmospheric particulate matter in specific environments such as coastal regions and marshlands, at concentrations raging from 0.01 pg/m^3^ to 5 pg/m^3^. These compounds are likely derived from the microbial methylation of inorganic precursors [8]. A particularly concerning source of volatile organic arsenicals is informal electronic waste recycling, in which compounds like trimethylarsine oxide are released, potentially forming secondary pollutants in the atmosphere [8,25].

The association of arsenic with PM_2_._5_ facilitates its long-range atmospheric transport [17]. Research in Spain’s Doñana National Park shows that arsenic from industrial areas can be transported hundreds of kilometers via atmospheric circulation, polluting remote ecosystems [33]. This migration is accompanied by form transformation: the oxidation rate of As^III^ reaches 30–50% during transport, altering its toxicity [33]. Deposited arsenic on surfaces can be absorbed by plant leaves or accumulate through the soil–plant system, creating indirect exposure pathways for humans [9].

### 3.3. Particle Size-Dependent Deposition

The health implications of inhaled arsenic are critically dependent on the aerodynamic diameter of the carrier particles, which governs their deposition patterns within the respiratory tract and subsequent systemic bioavailability [4]. Industrial processes like mining and smelting generate a spectrum of particle sizes. Coarse particles (>10 μm) produced from crushing and grinding operations primarily deposit in the nasopharyngeal and tracheobronchial regions, where they are largely cleared by mucociliary action or swallowed. In contrast, fine (<2.5 μm) and ultrafine (<1 μm) particles from smelting and semiconductor manufacturing penetrate deep into the pulmonary alveoli, where systemic absorption is most efficient [4,26]. The size of the particle also influences its physicochemical properties. For example, coal fly ash, rich in As (V), exhibits particle size-dependent solubility and lung retention [4,26]. Smaller particles (<2 μm) from mining operations not only carry higher metal concentrations but also demonstrate greater bioavailability, thereby amplifying toxicity risks [4,26]. Moreover, fine and ultrafine particles have a greater potential for long-distance travel, contributing to widespread environmental contamination far from the original emission source [26]. The deposition mechanisms also differ: ultrafine particles (<0.1 μm) primarily deposit via Brownian diffusion, while those between 0.1 and 2.5 μm settle through sedimentation, both of which can prolong lung retention time and enhance the dissolution and absorption of arsenic [3,4]. This size-dependent behavior underscores why PM_2_._5_-bound arsenic poses a far greater health risk than larger particles and highlights the necessity of incorporating particle-size-specific analyses into occupational and environmental risk assessments [3,4].

### 3.4. Intervention Effects on Temporal Trends

Long-term monitoring data provide compelling evidence that concerted pollution control measures (e.g., flue gas desulfurization, baghouse filters, etc.) can significantly attenuate airborne arsenic concentrations [32]. Since China implemented the Air Pollution Prevention and Control Action Plan in 2013, arsenic emissions from key industries have decreased by over 30%, and annual average arsenic concentrations in industrial PM_2_._5_ have dropped by 6.5% [34,35]. European experience indicates that industrial production cuts during economic crises and application of best available techniques (BAT) can reduce arsenic emissions by over 40%, but improvements are reversible [36]. Pollution levels may rebound when production resumes. For example, in Greece during the 2008 financial crisis, industrial arsenic emissions dropped by 42%, but rebounded to 95% of pre-crisis levels by 2015 [37,38]. India’s case warns of environmental costs from unregulated development. Despite emission regulations, coal-related arsenic pollution in India has risen significantly, with atmospheric arsenic concentrations increasing at an annual rate of 6.5% between 2005 and 2015, far exceeding the global average [18,35]. This trend, combined with electronic waste dismantling in some Southeast Asian countries, poses new challenges for global arsenic pollution [9].

## 4. Biological Transport Mechanisms and Metabolic Fate

While ingestion through drinking water remains the predominant exposure route for the general population, inhalation represents a toxicologically distinct pathway that bypasses first-pass hepatic metabolism and leads to prolonged retention in lung tissue [39]. Despite a lower pulmonary bioavailability (~30%) than gastrointestinal absorption (~60%), arsenic’s biological half-life in the lungs persists for years, compared to mere hours in the bloodstream [39]. This protracted residence time is attributed to the sequestration of arsenic-laden particles by alveolar macrophages and epithelial cells, creating a persistent source of internal exposure that can lead to chronic local and systemic effects [39].

The deep penetration of fine particulate arsenic into the alveolar region facilitates rapid dissolution in the pulmonary surfactant layer and efficient absorption into the systemic circulation [39]. Consequently, inhaled arsenic delivers a more direct and potent dose to sensitive extra-pulmonary organs, such as the heart and brain, with studies indicating that organ concentrations can be more than double those achieved via oral exposure at equivalent doses [39]. This pharmacokinetic profile helps explain the disproportionate risk of lung cancer observed in occupationally exposed populations (e.g., smelter workers) compared to populations exposed primarily through drinking water, even when total arsenic intake is comparable [39].

Following gastrointestinal and/or pulmonary absorption, inorganic arsenic undergoes a complex process of systemic distribution, biotransformation (primarily methylation), and elimination [4,9,39]. It exhibits a particular affinity for keratin-rich tissues, including skin, hair, and nails, making these valuable biomarkers of exposure [13]. The primary route of elimination for absorbed arsenic is urinary excretion of its metabolites incuding inorganic arsenic (iAs), monomethylarsonic acid (MMA), and dimethylarsinic acid (DMA). The relative proportions of these compounds are used as indicators of an individual’s metabolic capacity and susceptibility [40,41].

A growing body of epidemiological evidence has established positive associations between ambient airborne arsenic exposure and a spectrum of adverse health outcomes, particularly cardiovascular effects such as myocardial infarction, coronary events, impaired heart rate variability, hypertension, systemic oxidative stress, and inflammation [42,43,44]. Furthermore, chronic environmental exposure of humans to arsenic has been consistently linked to an increased risk of cancer and a wide array of other health impacts, with emerging evidence pointing to significant neurodevelopmental impairments [42,43,44]. The subsequent sections will delve into the molecular mechanisms underpinning these organ-specific pathologies.

## 5. Molecular Mechanisms of Arsenic Toxicity

The toxicity of airborne arsenic is not attributable to a single pathway but rather emerges from a cascade of interconnected molecular events predominantly driven by oxidative stress [45,46,47]. This section delineates the core mechanisms through which arsenic, particularly in its trivalent form (As^III^), disrupts cellular homeostasis, leading to widespread damage.

### 5.1. Multiple Pathways of Reactive Oxygen Species (ROS) Generation

**Figure 2:**
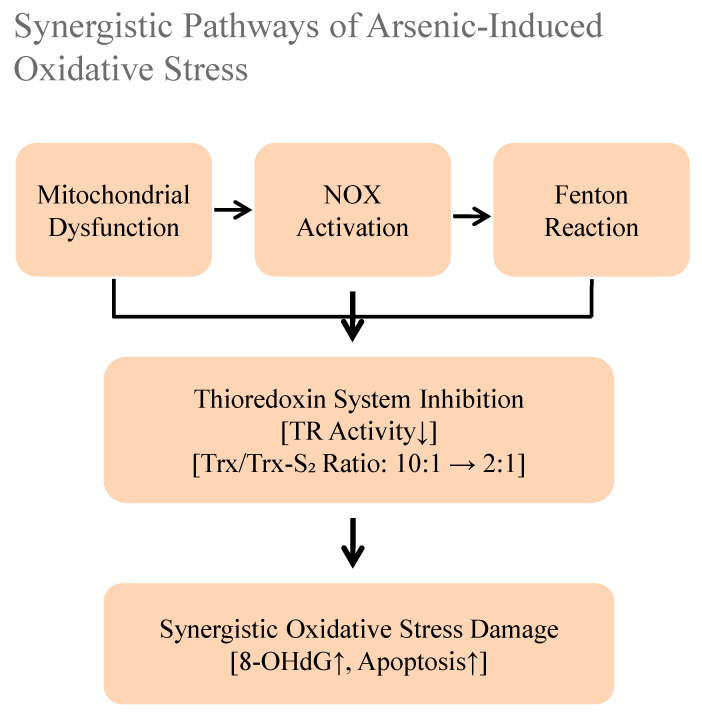
The pathways of reactive oxygen species (ROS) generation from arsenic in vivo [45,46,47]. The upward arrow represents triggering or activation, while the downward arrow signifies inhibition.

The excessive generation of reactive oxygen species (ROS) constitutes a critical initiating event in arsenic toxicity driven by multiple synergistic pathways (Figure 2). Arsenic (As^III^) strongly blocks mitochondrial respiratory chain complexes, such as Complex I [45]. This causes electrons to leak and form superoxide. It concurrently activates membrane-bound NADPH oxidases (NOX) and exacerbates the Fenton reaction by complexing with intracellular iron, catalyzing the production of highly damaging hydroxyl radicals [45,46,47]. Furthermore, arsenic directly incapacitates key antioxidant systems, most notably by inhibiting thioredoxin reductase (TR), thereby crippling the thioredoxin system and shifting the intracellular environment toward profound oxidative stress. The convergence of these pathways creates a self-amplifying cycle of oxidative damage that underlies much of arsenic’s pathogenicity [45,46,47].

### 5.2. Collapse of Antioxidant Defense Systems

In parallel to inducing ROS production, arsenic systematically dismantles the cell’s antioxidant defense network [48,49,50]. The tripeptide glutathione (GSH) is rapidly depleted through complex formation with H_3_AsO_3_. Key antioxidant enzymes such as superoxide dismutase (SOD), catalase (CAT), and glutathione peroxidase (GPx) are inhibited due to arsenic’s modification of critical cysteine residues [48,49,50]. Perhaps most critically, arsenic disrupts the Nrf2-Keap1 pathway, the master regulatory response to oxidative stress. As^III^ binds to cysteine thiol groups on Keap1, forming reversible As–S bonds, preventing Nrf2 translocation, and blunting the induction of protective enzymes like heme oxygenase-1 (HO-1). This suppression results in a sustained state of oxidative stress, leaving cells chronically vulnerable [48,49,50].

### 5.3. Molecular Consequences of Oxidative Damage

The relentless oxidative assault orchestrated by arsenic manifests as extensive damage to all major classes of biomolecules, driving cellular dysfunction and death [51]. DNA damage is a hallmark consequence, where hydroxyl radicals avidly attack guanine bases to form 8-OHdG adducts and induce single-strand breaks, significantly elevating the mutational load and cancer risk [51,52].

Lipid peroxidation is another major outcome. ROS can attack polyunsaturated fatty acids in cellular membranes, generating reactive aldehydes like malondialdehyde (MDA). MDA, in turn, cross-links with membrane proteins, impairing the function of critical transporters like Na^+^-K^+^-ATPase. Protein function is also severely compromised [53]. Arsenic binding inactivates key metabolic complexes like pyruvate dehydrogenase, slashing ATP production. Furthermore, oxidative modification of proteins like α-synuclein promotes their aggregation, which is pathologically reminiscent of neurodegenerative diseases [54,55].

Organelle integrity is not spared. The endoplasmic reticulum (ER), under oxidative duress, activates the unfolded protein response (UPR), which can culminate in apoptosis [45,53,56]. Mitochondria, both a source and target of arsenic-induced ROS, suffer severe damage to their own DNA (mtDNA), which experiences a much higher frequency of oxidative lesions due to a lack of histone protection, leading to a catastrophic decline in energy production. This multi-target damage creates a vicious, self-amplifying cycle that ultimately leads to cell death, tissue damage, and disease pathogenesis [45,53,56].

## 6. Signaling Pathway Disorders

The widespread molecular damage induced by arsenic inevitably leads to the dysregulation of critical cellular signaling networks that govern cell fate, identity, and function [57,58,59]. This dysregulation provides the mechanistic link between initial oxidative insult and the development of specific pathologies [57,58,59].

### 6.1. Abnormal DNA Methylation

Arsenic exerts a profound and paradoxical influence on the epigenetic landscape, primarily through disrupting DNA methylation patterns. It depletes S-adenosylmethionine (SAM), the universal methyl donor. SAM is utilized for both the methylation and detoxification of arsenic, and its depletion leads to DNA hypomethylation [57]. This loss of methylation, particularly in repetitive genomic elements like LINE-1, compromises genomic stability and may activate proto-oncogenes [57,58,59].

Conversely, arsenic also induces localized promoter hypermethylation of specific tumor suppressor genes [59,60]. For example, the promoter of p16INK4a, a critical cell cycle regulator, showed a methylation rate of 60% in an arsenic-exposed population from Bangladesh, which was three times higher than in control groups [9]. This epigenetic silencing effectively disables a crucial brake on cell proliferation. Similarly, hypermethylation of death-associated protein kinase (DAPK) and microRNA promoters like miR-34a disrupts normal apoptosis and senescence programs [59,60].

These epigenetic alterations are not limited to the exposed individual; arsenic can cause hypermethylation at imprinting control regions (e.g., H19) in germ cells and placental tissue, which is associated with adverse developmental outcomes like low birth weight, suggesting a mechanism for its transgenerational effects [61,62].

### 6.2. Reprogramming of Histone Modifications

The pattern of covalent modifications on histone proteins is another key epigenetic target of arsenic [54]. It disrupts the delicate balance of covalent histone modifications, which in turn alters chromatin structure and gene expression. Arsenic inhibits histone deacetylases (HDACs) while simultaneously activating histone acetyltransferases (HATs), leading to a net increase in histone acetylation (e.g., H3K9ac). In neuronal cells, this hyperacetylation is associated with suppressed expression of memory-related genes [63,64,65].

The balance of histone methylation is also skewed. Arsenic reduces repressive marks like H3K27me3 by inhibiting the methyltransferase EZH2, while it increases activating marks like H3K4me3 [66,67,68]. In lung cancer cells, this shift results in a dramatic increase in H3K4me3 enrichment at the promoter of the oncogene c-Myc, fueling its transcription and driving proliferation [66,67,68]. Furthermore, arsenic induces the ubiquitination of histone H2A (H2Aub), which accumulates at sites of DNA damage (e.g., in human lung cells) [65]. While this is part of a normal DNA damage response, excessive H2Aub impairs the efficiency of DNA repair mechanisms. This collective reprogramming of the histone landscape fundamentally alters cellular identity and function, promoting a state conducive to carcinogenesis and other diseases [66,67,68].

### 6.3. Dysregulation of Non-Coding RNA Networks

The regulatory roles of non-coding RNAs (ncRNAs) represent a critical layer of arsenic’s toxic influence. Arsenic exposure remodels the microRNA (miRNA) expression profile, often promoting a pro-oncogenic environment [69,70,71]. For instance, it upregulates oncogenic miR-21, which targets and suppresses the tumor suppressor PTEN, leading to hyperactivation of the PI3K/AKT survival pathway and enhanced cell migration. Concurrently, it downregulates tumor-suppressive miRNAs like miR-200c, which relieves inhibition of the transcription factor ZEB1, a master key driver of epithelial–mesenchymal transition (EMT) [69,70,71].

Long non-coding RNAs (lncRNAs) are also involved. The expression of lncRNA MALAT1 is highly elevated in arsenic-transformed cells. MALAT1 acts as a competing endogenous RNA (ceRNA) or sponge, sequestering miR-200c and further enhancing ZEB1 expression, thereby stabilizing a pro-metastatic gene program [72,73]. Circular RNAs (circRNAs), a more recently discovered class of ncRNAs, are also implicated. For example, circ_0013597 is upregulated in arsenic-exposed hepatocytes and promotes angiogenesis by stabilizing VEGFA mRNA, potentially contributing to tumor progression. The coordinated dysregulation of these diverse ncRNA networks allows arsenic to exert widespread control over gene expression patterns that dictate cell survival, proliferation, and motility [72,73].

### 6.4. Transgenerational Epigenetic Effects

Perhaps one of the most concerning aspects of arsenic toxicity is its capacity to inflict health effects that transcend the exposed generation. This is particularly notable following oral exposure, such as through contaminated drinking water. Animal studies involving oral administration show that ingested arsenic causes systemic epigenetic changes in sperm and eggs [62,74]. These changes can pass to future generations, even if they are not directly exposed. Paternal exposure leads to hypomethylation at imprinting control regions in sperm DNA, which is associated with an increased incidence of metabolic disorders like insulin resistance in offspring [62,74]

In zebrafish models, ancestral arsenic exposure results in hundreds of differentially methylated sites in the sperm of F2 males, with these sites enriched in genes critical for development and signaling [62,74]. These epigenetic memories manifest in altered phenotypes in the unexposed descendants, including anxiety-like behaviors linked to hypermethylation of the *BDNF* gene promoter in the brain. This transgenerational inheritance mechanism suggests that the health impacts of environmental arsenic exposure may be far more pervasive and long-lasting than previously recognized, affecting the viability and health of future generations through stable epigenetic reprogramming of the germline [62,74].

## 7. Regulatory Disorders

The epigenetic and oxidative damage orchestrated by arsenic converges on the dysregulation of core signal transduction pathways that are fundamental to controlling cell growth, stress response, and inflammation [75,76].

### 7.1. Disorder of MAPK Signaling Network

Mitogen-activated protein kinase (MAPK) pathways respond to arsenic in a biphasic, dose-dependent manner. At low doses, arsenic promotes cell proliferation and survival by preferentially activating the Raf-MEK-ERK signaling axis. This is characterized by increased phosphorylation of MEK and ERK, leading to a elevated proliferation index in cells like keratinocytes [75,76,77].

In stark contrast, high-dose arsenic exposure activates the stress-responsive JNK and p38 MAPK pathways [75,76,77]. This activation, driven heavily by oxidative stress, triggers pro-apoptotic and inflammatory responses. In neuronal cells, sustained p38 activation leads to hyperphosphorylation of tau protein, mirroring a pathological feature of Alzheimer’s disease. Crosstalk exists between these pathways; ROS generated by arsenic can mediate cross-activation between ERK and JNK, a combination that may promote cellular transformation rather than outright death. This dose-dependent switching between pro-survival and pro-death signaling helps explain the dual role of arsenic as both a promoter and an inducer of carcinogenesis [75,76,77].

### 7.2. Dysregulation of PI3K/AKT/mTOR Signaling

The PI3K/AKT pathway, a central regulator of metabolism, growth, and survival, is profoundly affected by arsenic in a time-dependent manner. Short-term exposure can activate AKT, providing a transient survival signal [70,78]. However, long-term exposure leads to chronic, dysregulated AKT activation, often due to oxidative inactivation of its negative regulator, PTEN. This persistent AKT signaling is a common feature in arsenic-associated cancers [70,78].

A key downstream effector of AKT is the mechanistic target of rapamycin complex 1 (mTORC1), a master regulator of protein synthesis and cell growth [56,78]. Arsenic hyperactivates mTORC1 by inhibiting the GAP activity of the TSC2 complex. In renal cells, this leads to cellular hypertrophy and increased protein synthesis. This signaling node also critically regulates autophagy [56,78]. While low-dose arsenic may induce protective autophagy, high-dose exposure potently inhibits autophagic flux via mTORC1 activation, preventing the clearance of damaged organelles and proteins and thus exacerbating cellular damage. The dysregulation of this axis integrates survival signals with metabolic control, playing a pivotal role in arsenic-induced adaptive changes and pathology [56,78].

### 7.3. Activation of NF-κB Inflammatory Signaling

The transcription factor NF-κB is a primary mediator of inflammatory responses and is chronically activated by arsenic, creating a persistent state of low-grade inflammation. Arsenic induces the phosphorylation and proteasomal degradation of IκBα, the inhibitor that sequesters NF-κB in the cytoplasm. This releases NF-κB dimers (e.g., p65/p50) to translocate into the nucleus [44,79].

Furthermore, As^III^ can directly bind to a cysteine residue on the p65 subunit of NF-κB, enhancing its DNA-binding affinity and transcriptional activity [44,80]. This leads to a massive upregulation of pro-inflammatory cytokines such as IL-6 and TNF-α. These cytokines then act on both the producing cells and neighboring cells to further activate NF-κB and recruit immune cells, establishing a self-perpetuating positive feedback loop known as the oxidative stress-inflammation cycle [44,80]. In the context of atherosclerosis (e.g., in ApoE^−/−^ mouse models), this process amplifies macrophage infiltration into vascular plaques and accelerates disease progression [44,80]. This chronic inflammatory microenvironment is a key contributor to the development of various arsenic-induced diseases, including cancer, cardiovascular disease, and diabetes [44,80].

### 7.4. Calcium Signaling and Cytoskeletal Regulation

Arsenic disruption of calcium homeostasis affects cellular functions [15,73]. Activated NF-κB induces the expression of pro-inflammatory factors such as TNF-α and IL-6, forming a positive feedback loop of oxidative stress–inflammation. In an atherosclerosis model, arsenic exposure increases TNF-α levels in plaques by 3-fold and macrophage infiltration by 50% [44].

Arsenic disrupts the precise spatiotemporal control of intracellular calcium (Ca^2+^), a ubiquitous second messenger. It promotes Ca^2+^ release from internal stores like the endoplasmic reticulum while also inhibiting Ca^2+^ extrusion pumps, leading to a sustained elevation of cytosolic Ca^2+^ levels. This calcium dyshomeostasis has myriad consequences [81].

Elevated cytosolic Ca^2+^ can aberrantly activate calmodulin (CaM) and downstream enzymes like phosphodiesterases and calcineurin, disrupting cyclic nucleotide signaling and gene expression [81]. In cardiomyocytes, this contributes to calcium overload and arrhythmias. Furthermore, calcium is essential for the dynamic regulation of the cytoskeleton [81]. Arsenic impairs the function of calcium-dependent actin-severing proteins like gelsolin, disrupting the normal assembly and disassembly of actin microfilaments. This alters cell morphology and reduces motility, but paradoxically may enhance invasiveness in cancer cells by promoting the expression of matrix metalloproteinases (MMPs). The disruption of Ca^2+^ signaling thus represents a fundamental mechanism through which arsenic alters everything from electrical excitability to cell shape and migration [81].

## 8. Organ-Specific Toxicity Mechanisms

**Figure 3:**
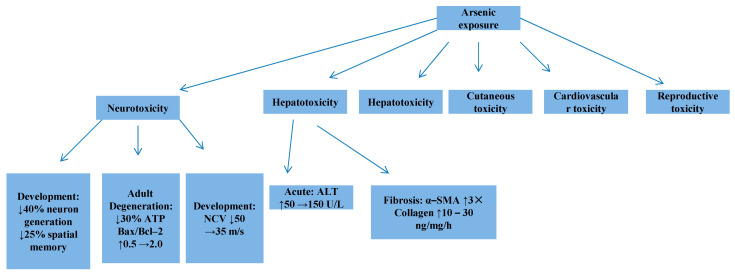
Organ-specific toxicity mechanisms for arsenic [77,82,83,84,85,86,87,88,89,90,91,92,93,94,95,96]. The upward arrow represents triggering or activation, while the downward arrow signifies inhibition.

The systemic distribution of arsenic following inhalation exposure culminates in distinct pathologies across major organ systems (Figure 3). Although the fundamental molecular drivers are shared, including oxidative stress, epigenetic dysregulation, and the disruption of signaling pathways, their specific manifestations are critically shaped by the unique cellular environments and metabolic capacities of different tissues [77,82,83,84,85]. This section synthesizes the mechanisms underpinning arsenic’s multi-organ toxicity, moving beyond a mere cataloging of effects to highlight integrative pathological processes.

### 8.1. Neurotoxicity

Arsenic-induced neurotoxicity presents a dual challenge, impairing both developmental processes in the young and accelerating degenerative pathways in adults (Figure 3). During gestation, arsenic exposure disrupts key neurodevelopmental signaling, notably the Shh pathway, leading to a significant reduction (~40%) in forebrain neuron generation [77,82,83,84,85]. Rodent models demonstrate that in utero exposure depletes hippocampal neural stem cells, resulting in measurable deficits in spatial memory tasks [77,82,83,84,85]. In mature organisms, chronic exposure triggers neuronal apoptosis through multifaceted mechanisms. These include the inhibition of mitochondrial energy production, which reduces ATP by 30 percent; the activation of pro-apoptotic JNK signaling, which elevates the Bax-to-Bcl two ratio; and the promotion of toxic alpha synuclein oligomerization, a hallmark of proteinopathic neurodegeneration [51,77,78]. Furthermore, arsenic preferentially targets peripheral sensory neurons to cause axonal degeneration and demyelination. These pathologies manifest as slowed nerve conduction velocity and the characteristic glove and stocking sensory loss, thereby linking molecular damage directly to clinical neuropathy [77,82,83,84,85].

### 8.2. Hepatotoxicity

The liver, as a primary site of arsenic metabolism, is particularly vulnerable [86,87,88]. Acute high-dose exposure induces rapid centrilobular necrosis, mediated by mitochondrial permeability transition pore opening and catastrophic energy collapse, evidenced by precipitous rises in serum ALT. Chronic exposure drives a more insidious pathology centered on progressive fibrosis. Arsenic-activated Kupffer cells release pro-fibrotic mediators like TNF-α and TGF-β1, which in turn activate hepatic stellate cells, culminating in a 3-fold increase in collagen synthesis [86,87,88]. The carcinogenic endpoint involves a sinister synergy between epigenetic silencing (e.g., hypermethylation of the p53 promoter) and persistent oxidative stress, which promotes hepatocyte immortalization. Epidemiological data from endemic regions, such as Taiwan’s blackfoot disease area, confirm a near 3-fold increase in liver cancer incidence, often with a distinct mutational signature involving TERT and CTNNB1 [86,87,88].

### 8.3. Nephrotoxicity

The kidney’s role in concentrating and excreting arsenic makes it a key target for damage primarily affecting the proximal tubules [89,90,91]. Arsenic directly inhibits Na^+^-K^+^-ATPase activity by binding to critical cysteine residues, disrupting reabsorptive function and leading to Fanconi syndrome, characterized by glucosuria and aminoaciduria. The correlation between urinary arsenic levels and biomarker elevation (e.g., α1-microglobulin) underscores this dose-dependent tubular injury [89,90,91]. Concurrently, arsenic disrupts glomerular integrity by activating the complement cascade, increasing basement membrane permeability and resulting in microalbuminuria. Over time, these insults precipitate a transition to chronic kidney disease via epithelial–mesenchymal transition (EMT) in tubular cells, marked by loss of E-cadherin and gain of vimentin, driving renal fibrosis and collagen deposition [89,90,91].

### 8.4. Cutaneous Toxicity

Dermatological manifestations provide visible markers of chronic arsenic exposure [92,93]. The classic raindrop pigmentation arises from arsenic’s dual action on melanocytes: paradoxically inhibiting tyrosinase activity while simultaneously stimulating melanocyte proliferation and melanosome transfer [92,93]. Hyperkeratosis, particularly on palms and soles, results from aberrant activation of the Wnt/β-catenin pathway, accelerating keratinocyte proliferation. The most severe dermal outcome is carcinogenesis [92,93]. The progression from Bowen’s disease to invasive squamous cell carcinoma is fueled by a combination of high-frequency HRAS mutations and epigenetic silencing of tumor suppressors like p16. The exceedingly high odds ratio (OR > 15) for skin cancer in exposed populations highlights the potent carcinogenicity of dermal arsenic accumulation [92,93].

### 8.5. Cardiovascular Toxicity

Arsenic wreaks havoc on the cardiovascular system by targeting endothelial homeostasis [44,94]. It quenches nitric oxide (NO) bioavailability by inhibiting endothelial nitric oxide synthase (eNOS) while stimulating the vasoconstrictor endothelin-1 (ET-1), creating a pro-hypertensive state [44,94]. Clinically, this manifests as impaired vascular dilation and widened pulse pressure. Arsenic also accelerates atherosclerosis by oxidizing LDL to its pro-atherogenic form (ox-LDL) and upregulating the scavenger receptor CD36 on macrophages, enhancing foam cell formation [44,94]. Histological studies of plaques from exposed individuals reveal dense macrophage infiltration. Direct cardiotoxicity is evidenced by arsenic’s inhibition of mitochondrial complex IV, impairing cardiac contractility and ultimately leading to dilated cardiomyopathy and reduced ejection fraction in experimental models [44,94].

### 8.6. Reproductive Toxicity

Arsenic’s reproductive toxicity exhibits stark gender specificity and extends transgenerationally via epigenetic inheritance. Studies administering doses such as 50 mg/kg sodium arsenite in rodent models (e.g., mice) have observed these effects in offspring after only one to two generations of exposure [74,95,96]. In males, it disrupts spermatogenesis, reducing sperm count and increasing morphological abnormalities by interfering with testosterone synthesis and meiotic fidelity, resulting in elevated sperm DNA fragmentation [74,95,96]. In females, it diminishes ovarian reserve by inhibiting granulula cell proliferation, shortening the reproductive window and elevating the risk of spontaneous abortion. Most critically, arsenic readily crosses the placental barrier, impairing trophoblast function and placental angiogenesis, which is strongly associated with low birth weight, as demonstrated in rodent models where maternal exposure to doses such as 10 ppm sodium arsenite via drinking water resulted in significant placental transfer [74,95,96]. As discussed in Section 6.4, these effects are not confined to the directly exposed generation; epigenetic reprogramming of germ cells allows arsenic-induced phenotypes, such as metabolic and neurobehavioral abnormalities, to be transmitted to subsequent, unexposed generations.

## 9. Health Risk Assessment and Regulatory Challenges

The translation of mechanistic toxicology into public health policy remains fraught with challenges, primarily due to outdated risk models and inadequate regulatory frameworks that fail to capture the complexity of arsenic toxicity.

### 9.1. Controversies in Carcinogenic Risk Assessment Models

The prevailing Linear No-Threshold (LNT) model, used by agencies like the U.S.’s EPA, estimates cancer risk from infinitesimally low doses by extrapolating linearly from high-exposure occupational data [39,97].

This model derives an inhalation Unit Risk Factor that predicts 43 excess lung cancers per 100,000 individuals from lifetime exposure to 1 μg/m^3^. However, a growing body of evidence from low-dose epidemiology (<0.1 μg/m^3^) suggests this model overestimates actual risk by 2- to 3-fold [39,97]. The LNT model does not include known nonlinear processes, such as metabolic saturation at certain doses [39,97]. It also ignores how low doses can activate stress responses and DNA repair [39,97]. These omissions lead to overestimation. Threshold models, which propose a No-Observed-Adverse-Effect Concentration (NOAEC), are gaining traction based on evidence that epigenetic changes and in vitro cell transformation exhibit clear dose thresholds, below which significant biological effects are absent [39,97].

### 9.2. Current Regulatory Standards and Limitations

As shown in Table 1, a comparative analysis of international regulations reveals a fragmented and often inadequate approach [98,99,100]. The EU’s non-binding target value (6 ng/m^3^ in PM_10_) and the U.S.’s lack of a national ambient standard leave large populations unprotected. While China has adopted a strict legal limit (6 ng/m^3^), rampant non-compliance in industrial and e-waste recycling zones, where arsenite exposure levels can exceed 200 ng/m^3^, renders the standard ineffective. The WHO’s risk-based guideline, while scientifically rigorous, does not account for cumulative exposure from other routes like water and diet, a critical flaw given that inhalation often constitutes a minor fraction of total intake in the most vulnerable, arsenic-endemic populations [98,99,100].

### 9.3. Scientific Challenges in Standard Setting

Three major gaps undermine current regulatory efforts. First, standards regulate airborne arsenic in isolation, ignoring the aggregate risk from concurrent exposure via drinking water, food, and soil, leading to a significant underestimation of total body burden and health risk. Second, children and pregnant women exhibit heightened susceptibility due to developmental vulnerabilities and immature detoxification systems, yet no standards incorporate safety factors to protect these groups specifically. Regulating total arsenic, rather than distinguishing between highly toxic inorganic species (e.g., As(OH)_3_) and relatively benign organic forms, creates a distorted risk landscape. It overestimates the risk from dietary sources like seafood while underestimating the threat from industrial emissions rich in bioavailable As(OH)_3_ [42,95,99].

### 9.4. Biological Monitoring and Risk Early Warning

Moving beyond environmental concentration measurements, biomarker-based strategies offer a path toward personalized risk assessment [40,52,59]. Speciated urinary arsenic (e.g., elevated %iAs) provides a direct measure of internal dose and metabolic competence, predicting disease risk. Long-term exposure is reliably captured in nail and hair arsenic content (e.g., >1 μg/g indicating chronic exposure) [100]. Effect biomarkers like 8-OHdG (DNA oxidation) and leukocyte p16 promoter methylation offer a glimpse into early biological damage, often years before clinical symptoms manifest [40,52,59]. Furthermore, susceptibility biomarkers, such as genetic polymorphisms in AS3MT and Nrf2, can identify genetically vulnerable individuals, paving the way for targeted monitoring and intervention strategies [40,52,59].

## 10. Future Research Directions

Bridging the identified knowledge gaps requires a concerted shift toward innovative methodologies and interdisciplinary integration.

Priority must be given to defining the dose–response curve for low-dose, chronic inhalation exposure using sensitive omics technologies. Long-term animal inhalation studies coupled with single-cell epigenomic profiling (e.g., scATAC-seq) of target tissues like lung and brain will elucidate the precise thresholds for epigenetic dysregulation. Furthermore, multi-generational model studies are essential to decipher the mechanisms of epigenetic inheritance and identify germline-specific differentially methylated regions (DMRs) that serve as biomarkers for transgenerational risk [57,62]. The future of exposure assessment lies in granular, real-time data, making it essential for capturing the impact of toxins like arsenic, which affects ovarian reserve function by inhibiting granulosa cell proliferation [74]. We should use networks of sensitive graphene sensors that detect arsenic at 0.1 ng/m^3^, as such graphene-based sensors have demonstrated capability for real-time arsenic detection with remarkable sensitivity [102,103,104]. These sensors, connected to 5G and IoT systems, can map pollution in real time and identify its sources. Complementing this, next-generation personal wearable monitors that track inhaled dose, respiratory rate, and activity patterns will provide unprecedented resolution in individual exposure assessment, moving beyond static ambient measurements [73,105].

To overcome the current regulatory silos, we must develop and validate integrated PBPK/PD models that simulate arsenic pharmacokinetics and pharmacodynamics across the life course, incorporating genetic (AS3MT status), physiological (pregnancy), and nutritional (folate levels) variables. These models should aggregate exposure from all relevant routes (air, water, diet, dust) to generate a holistic risk estimate, providing a scientifically robust foundation for setting truly protective public health standards [40,97].

## 11. Conclusions

This review synthesizes the substantial and complex body of evidence on the toxicology of airborne arsenic, delineating a pathway from molecular insult to public health crisis. I have established that arsenic’s toxicity is not a simple story of chemical poisoning but a multifaceted saga involving oxidative stress, epigenetic reprogramming, and signaling network disruption, culminating in organ-specific pathologies from neurodevelopmental deficits to carcinogenesis. Our analysis concludes that existing regulatory frameworks are fundamentally inadequate to address this complexity. The rigid LNT model overestimates low-dose risks, while existing standards fail to protect the most vulnerable and ignore the reality of cumulative, multi-route exposure. The way forward demands a paradigm shift. Future research must prioritize defining the nonlinear, low-dose mechanisms and transgenerational consequences. Risk assessment must evolve to embrace integrated, biomonitoring informed models that account for total human exposure. Public health efforts should use new technology to control pollution sources. They should also focus protection on the most vulnerable people. Only through such a comprehensive and mechanistic approach can we hope to mitigate the global health burden imposed by persistent airborne arsenic pollution.

## Figures and Tables

**Table 1:** Comparisons of international and regional standards [98,99,100].

Standard/Region	Limit/Guideline	Legal Status	Notes
European Union (2004/107/EC)	Annual target: 6 ng/m^3^ (As in PM_10_)	Non-binding	2017 monitoring: Only 7/645 sites exceeded the limit (max: 550 ng/m^3^ near Bor copper plant, Serbia).
United States	No federal air arsenic standard	/	/
OSHA	Workplace PEL-TWA: 10 μg/m^3^ (inorganic As)	Binding (occupational)	Does not cover general public exposure.
California OEHHA	Chronic REL-TWA: 0.015 μg/m^3^ (developmental toxicity)	Non-binding (advisory)	Health-based reference exposure level.
China (GB3095-2012) [101]	Annual limit: 6 ng/m^3^	Binding	E-waste dismantling areas measured up to 200 ng/m^3^ (33× above limit).
WHO	Unit Risk Factor (URF): 1.5 × 10^−3^ (μg/m^3^)^−1^ (lung cancer risk)	Guideline	Corresponds to 6.6 ng/m^3^ for 1:10^5^ lifetime risk; high-pollution areas (≥30 ng/m^3^) should assess inhaled–oral dose equivalence.

## Data Availability

No new data were created or analyzed in this study. Data sharing is not applicable to this article.
